# Therapeutic management of in-stent thrombosis after thoracic endovascular aortic repair for blunt thoracic aortic injury in a coronavirus disease 2019 patient

**DOI:** 10.1016/j.jvscit.2023.101297

**Published:** 2023-08-15

**Authors:** Karen van Rijn, Abbey Schepers, Rutger W. van der Meer, Carla S.P. van Rijswijk, Jan van Schaik, Joost R. van der Vorst

**Affiliations:** a Department of Vascular Surgery, Leiden University Medical Centre, Leiden, The Netherlands; b Department of Radiology, Leiden University Medical Centre, Leiden, The Netherlands

**Keywords:** In-stent thrombosis, Thoracic endovascular aortic repair

## Abstract

A 27-year-old man underwent thoracic endovascular aortic repair for blunt thoracic aortic injury. Fourteen months later, he presented with intermittent paraplegia, congestive heart failure, and a decline of kidney function as a result of high-grade aortic stenosis caused by in-stent thrombosis. He had a concurrent infection with coronavirus disease 2019. The patient was successfully treated using axillofemoral bypass, followed by stent relining 2 weeks later. The possible risk factors and the optimal therapeutic approach for in-stent thrombosis remain unknown, because only a limited number of cases describing this rare complication have been reported.

Blunt thoracic aortic injury (BTAI) is a rare, but disastrous, finding after high energy deceleration trauma.^1^^,^^2^ Thoracic endovascular aortic repair (TEVAR) is preferred over open surgery when feasible, considering the lower perioperative mortality and morbidity.^3^ A rare complication, reported only in a limited number of cases, is symptomatic stenosis and occlusion.^4^ The best treatment has not yet been determined. We report the successful treatment of a patient with coronavirus disease 2019 (COVID-19) who developed an acute subtotal occlusion of an aortic endograft due to in-stent thrombosis >1 year after stent graft placement for BTAI and discuss the available literature. The patient provided written informed consent for the report of his case details and imaging studies.

## Case report

**Fig 1:**
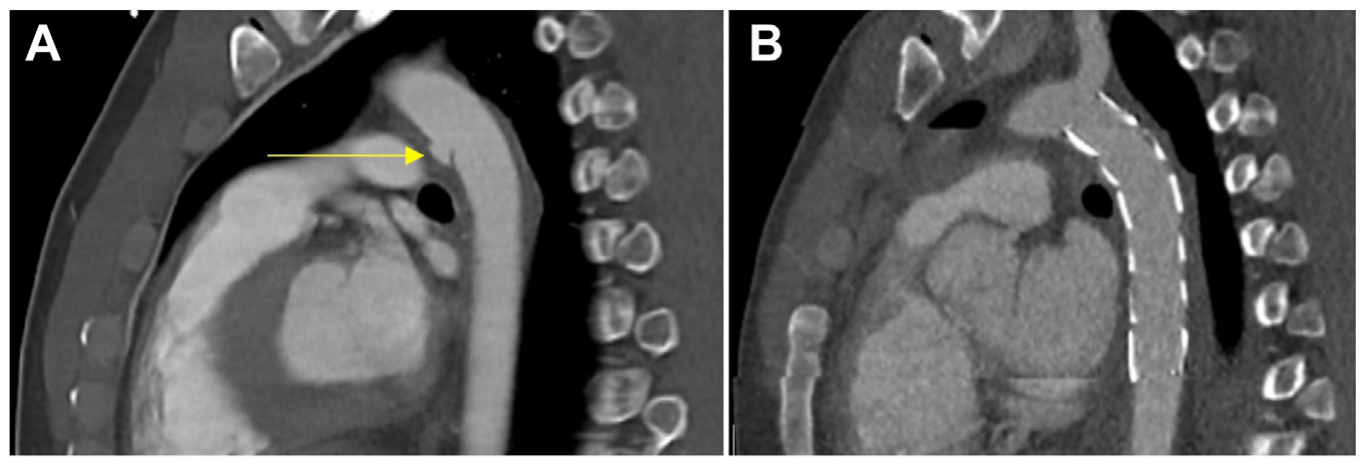
**A,** Computed tomography angiography (CTA) revealing a pseudoaneurysm and intimal tear at Harvey's ligament (*yellow arrow*). **B,** CTA after thoracic endovascular aortic repair (TEVAR).

A 27-year-old man presented to the emergency department after a fall from 10 meters. His injuries included multiple fractures of the pelvis and extremities. He also experienced a grade III BTAI (Fig 1, *A*), a pseudoaneurysm at Harvey's ligament, which was treated emergently with TEVAR using a 24-mm × 117-mm Zenith TX2 stent graft (Cook Medical Inc), because it is readily available at our center. The stent graft was placed distal to the left subclavian artery to cover the pseudoaneurysm without complications (Fig 1, *B*). The patient made a complete functional recovery and was prescribed antiplatelet therapy (aspirin) for life.

**Fig 2:**
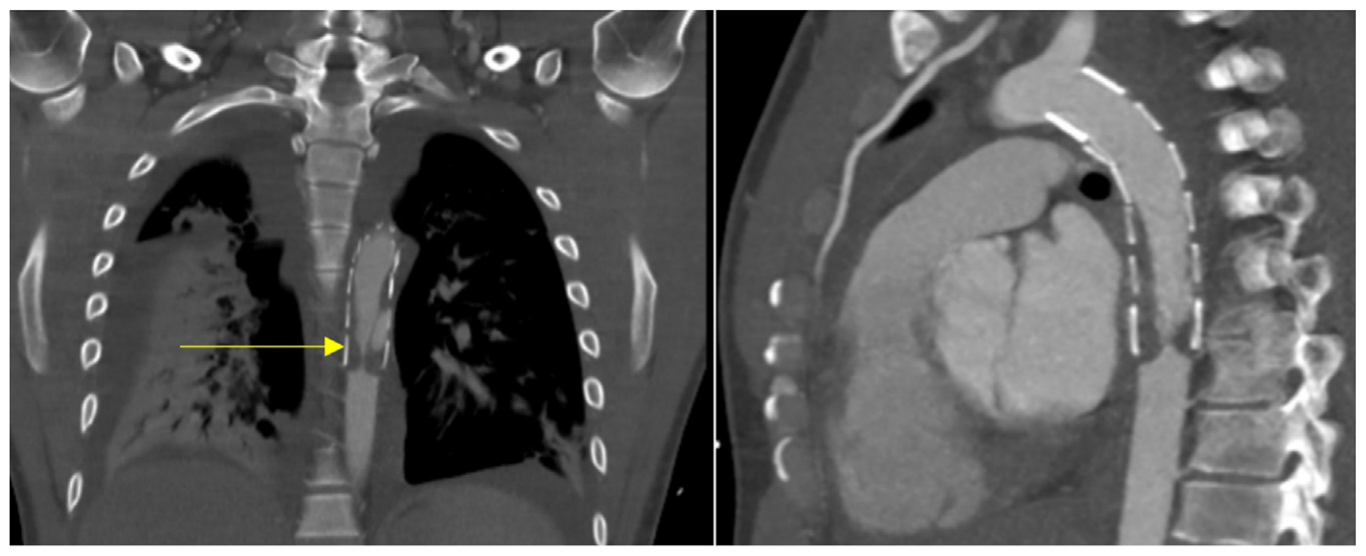
Computed tomography angiography (CTA) revealing circular wall thrombus of distal part of thoracic aortic endograft (*yellow arrow*) resulting in subtotal occlusion. Bilateral lung consolidation consistent with coronavirus disease 2019 (COVID-19) infection also visible.

At 14 months after surgery, he experienced an acute 4-hour period of a loss of sensory and motor function of both legs, followed by intermittent claudication and shortness of breath. He presented to the hospital 3 days later. Against medical advice, the patient had discontinued the use of antiplatelet therapy 6 months prior. Examination revealed hypertension (220 mm Hg systolic pressure). Blood tests revealed decreased kidney function (creatinine, 128 μmol/L; previously, 55 μmol/L) and congestive heart failure (pro-brain natriuretic peptide, 1541 ng/L). Computed tomography angiography (CTA) showed high-grade in-stent stenosis of the distal part of the thoracic endograft (Fig 2), resulting in clinically relevant malperfusion of the lower extremities, kidneys, and spinal cord. No concomitant venous thromboses were discovered. Additionally, the patient tested positive for COVID-19 at presentation (despite two vaccinations) and required noninvasive ventilation for adequate blood oxygen saturation. He was admitted to the intensive care unit and treated with anticoagulation therapy (intravenous heparin), antihypertensive agents (calcium channel blocker and a loop diuretic), and a statin for plaque stabilization. The blood test results were consistent with a prothrombotic milieu, which has also been described with COVID-19 infection in recent literature, including an increased D-dimer level (2600 ng/mL) and a twofold increase in fibrinogen.^5^^,^^6^

Considering the respiratory status of our patient, the initial treatment plan was to postpone acute surgery. However, 2 days after admission, the patient was intubated because of progressive respiratory failure. His kidney function then started to decline, and restoration of distal flow to the abdominal aorta was indicated to prevent further deterioration. Primary stenting was thought to include a high risk of distal embolization because the thrombus was expected to be at least in part of recent nature. It was reasoned that “buying time” to stabilize the thrombotic mass would allow for a safer stenting option in the future, if needed. Therefore, right-sided axillofemoral bypass surgery (8-mm × 70-cm heparin-bonded graft; Propaten vascular graft; W.L. Gore & Associates) was performed. Following surgery, the patient’s systolic blood pressure decreased to 160 mm Hg and his serum creatinine decreased to 63 μmol/L, indicating afterload reduction and better renal perfusion. The patient was extubated 1 day postoperatively. Intravenous heparin was switched to double antiplatelet therapy.

**Fig 3:**
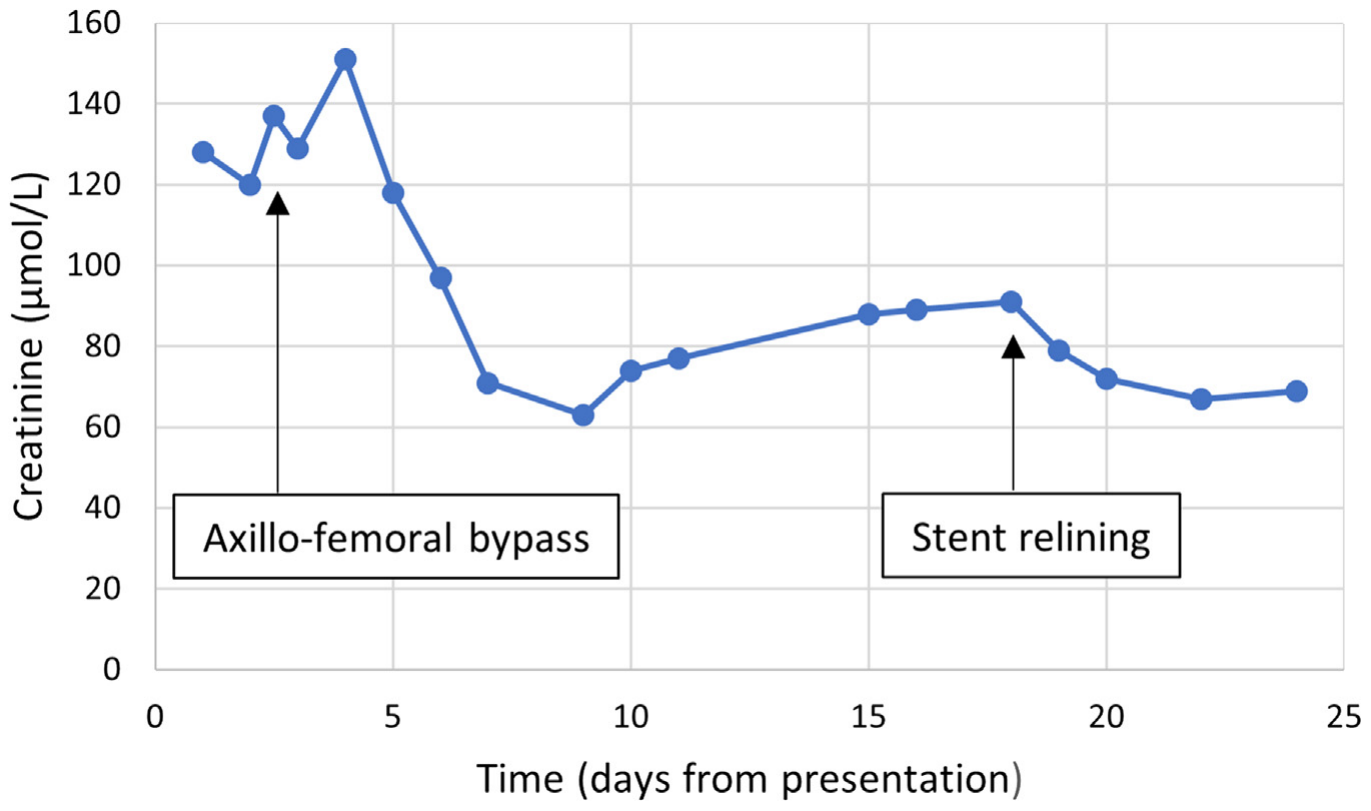
Graph representing kidney function expressed in creatinine levels over time with therapeutic interventions marked along the *x* axis.

**Fig 4:**
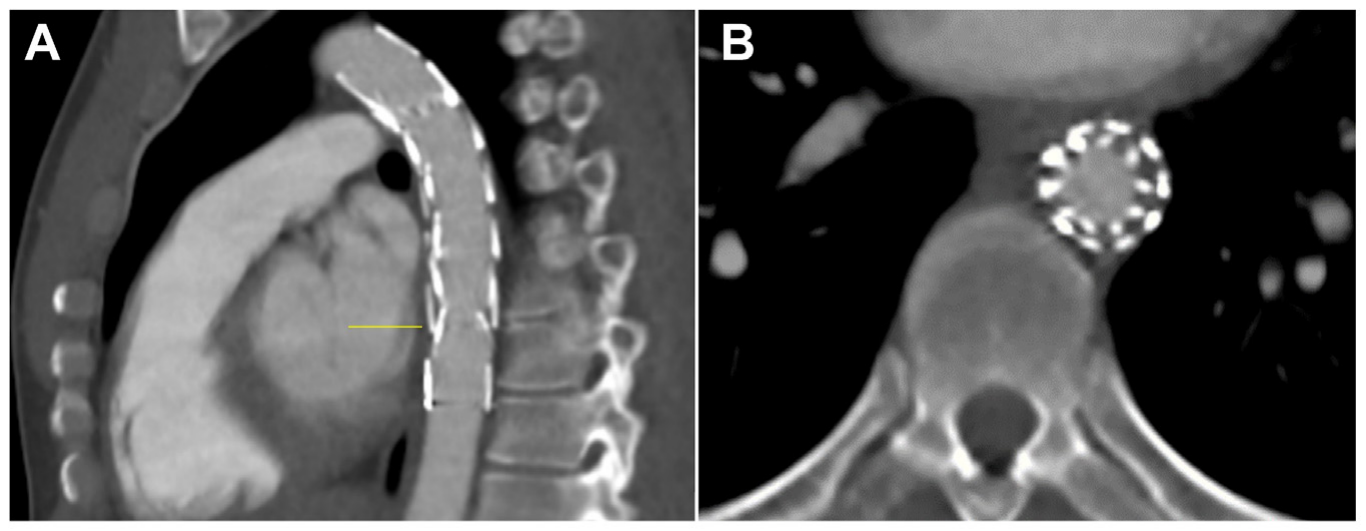
Computed tomography angiography (CTA) after relining of thoracic endovascular aortic repair (TEVAR). **A,** Sagittal plane. **B,** Axial plane at distal end of TEVAR (level of *yellow line* in Fig A) showing thrombus compressed behind the overlapping stent graft.

In the subsequent days, the patient remained hypertensive with systolic blood pressure of 160 to 180 mm Hg, despite multiple antihypertensive drugs. This was attributed to persistent renal hypoperfusion with activation of the renin-angiotensin system as a result. The kidney function again started to decline, with creatinine levels increasing to 91 μmol/L (Fig 3). Ultrasound revealed a patent bypass and flow in both renal arteries. The right-sided ankle to brachial pressure index measured 0.51, and the patient experienced severe intermittent claudication. Therefore, contrast-enhanced radiographically guided relining of the thoracic stent graft was performed to cover the in-stent thrombosis (24 mm × 112 mm; Valiant Captivia; Medtronic Vascular; Fig 4). To prevent distal embolization, no balloon dilatation was performed in the middle overlap zone to lower the risk of thrombus dislodgement. The procedure was uneventful and without thromboembolic complications. The patient was discharged from the hospital with a normalized blood pressure, increasing kidney function (creatinine, 69 μmol/L), and no intermittent claudication. Postoperative CTA after 1 month revealed successful relining and a false aneurysm on the proximal anastomosis of the axillofemoral bypass. The false aneurysm was successfully treated using thrombin injection during balloon occlusion of the axillary artery to prevent embolization. Explantation of the still-patent axillofemoral bypass was performed without complications after 3 months of recovery. At 9 months after stent relining, the patient was in good condition, and the ankle to brachial pressure index measured 1.0.

## Discussion

In the present case report, we describe the treatment of a late-onset subtotal occlusion of a thoracic stent graft after BTAI. Several therapeutic approaches have been previously described (Table). Open reconstruction was successfully performed in four cases.^8^^,^^13^^,^^15^^,^^16^ In one case reported by Reich et al,^10^ open surgery resulted in paraplegia. Because of the poor pulmonary status of our patient due to COVID-19, combined with the increased perioperative mortality and morbidity risk, we did not consider open reconstruction at presentation. Another approach described is axillofemoral bypass surgery. Although this is associated with low complication rates, in one of three cases, the bypass provided insufficient distal perfusion, which was the case in our patient.^7^^,^^11^^,^^16^ Urgent relining was an effective solution in three cases.^9^^,^^14^^,^^15^ In one case, it was complicated by distal microembolization, which was treated with low-molecular-weight heparin with no permanent morbidity.^9^ In the present case, when relining was performed subsequently to treat persistent renal malperfusion after axillofemoral bypass, no thromboembolic complications were observed. It is unknown in the present case whether an acute unstable thrombus susceptible to dislodgement or a slow-growing thrombus with lower embolic potential was present, in which case relining might have been considered as first treatment.^18^ Other treatment options, including suction thrombectomy, thrombolysis, and mechanical thrombectomy, were not considered because their efficacy in the case of patent flow was considered low and the risk of distal embolization high.

**Table:** Characteristics of 13 patients who experienced in-stent thrombosis after thoracic endovascular aortic repair for blunt thoracic aortic injury

Investigator	Patient age, years; sex	Oversizing, %	Antiplatelet therapy	Presentation	Treatment	Outcome
Alvarez et al,^7^ 2009	17; Male	30	ASA 100 mg/d	+11 Months, abdominal pain, intermittent paraplegia, heart failure	Axillofemoral bypass, followed by bypass from ascending to abdominal aorta	No complications; asymptomatic at 2 years
Marone et al,^8^ 2012	32; Male	26	ASA 100 mg/d	+24 Months, thrombus on follow-up CTA	Anticoagulation for 6 months, followed by open replacement	No complications; asymptomatic at 3 months
Marino et al,^9^ 2014	38; Male	27	ASA 100 mg/d	+6 Months, thrombus on follow-up CTA	Anticoagulation for 18 months, followed by relining	Bilateral distal microembolic lesions in legs; asymptomatic at 6 months
	32; Male	30	NR	+39 Months, headache, claudication	Relining	No complications; asymptomatic at 10 months
Reich et al,^10^ 2014	24; Male	4	ASA 81 mg/d	+14 Months, bilateral intermittent leg weakness	Open stent graft replacement	Paraplegia; no improvement at 1 year
Abdoli et al,^11^ 2017	29; Male	22	NR	+9 Months, limb ischemia, renal failure, gastrointestinal bleeding	Systemic heparin, followed by axillofemoral bypass	No complications; asymptomatic at 4 months; open replacement planned
García et al,^12^ 2018	NR	NR	NR	+12 Months, no information	Bypass from ascending to abdominal aorta	No complications
Liesdek et al,^13^ 2018	24; Male	NR	NR	+24 Months, acute paraplegia	Open stent graft replacement	No complications; paraplegia improving at 6 months
Hostalrich et al,^14^ 2019	15; Female	32	NR	+10 Months, bilateral leg weakness, renal failure	Relining	No complications; asymptomatic at 2 months
Martinelli et al,^15^ 2020	22; Male	20	NR	+6 Months, multiorgan failure, acute paraplegia	Relining	Persistent paraplegia, recurrent occlusion after 8 months, followed by open replacement
Beijer et al,^16^ 2021	21; Male	20	ASA 80 mg/d	+22 Months, acute paraplegia (5 days after car accident)	Axillofemoral bypass, followed by open replacement	Intestinal ischemic injury, kidney failure, paraplegia improving at 23 months
Chiu et al,^17^ 2022	19; Male	NR	ASA 81 mg/d	+8 Months, acute paraplegia	Systemic and local alteplase, followed by angioplasty	Multiorgan failure, followed by death
Present patient	27; Male	20	ASA 100 mg/d, discontinued after 8 months	+14 Months, intermittent paraplegia, kidney failure, heart failure (COVID-19)	Axillofemoral bypass, followed by stent relining	False aneurysm bypass; asymptomatic at 9 months

*ASA,* Acetylsalicylic acid; *COVID-19,* coronavirus disease 2019; *CTA,* computed tomography angiography; *NR,* not reported.

Previous case reports have suggested several causes for in-stent thrombus formation. Excessive oversizing can lead to graft infolding, causing turbulence of flow, leading to an increased risk of thrombus formation. In our case, the stent had an adequate oversize of 20%, and follow-up CTA showed proper sealing of the graft without infolding. One factor that could have contributed in the present case is that the patient had ceased to use antiplatelet therapy 6 months before readmission. Finally, recent literature suggests that COVID-19 infection increases the risk of venous and arterial thrombosis.^19^ It is suggested that COVID-19 induces endothelial injury and a cytokine storm, causing activation of coagulation factors and resulting in a hypercoagulable state.^5^^,^^6^ Other diseases causing hypercoagulability (eg, thrombophilia, myeloproliferative neoplasms, other malignancies) were excluded after consultation with a vascular internist.

## Conclusions

In-stent thrombosis after TEVAR is a rare, but life-threatening, complication, and treatment should be individualized. The hypercoagulability caused by active COVID-19 infection, combined with failure to comply with antiplatelet therapy, likely caused the formation of in-graft thrombosis in our patient. The literature on the best therapeutic approach is limited to case reports. In the present case, a two-step approach was chosen to minimize the risk to the patient.

## References

[1] Akhmerov A., DuBose J., Azizzadeh A. (2019). Blunt thoracic aortic injury: current therapies, outcomes, and challenges. Ann Vasc Dis.

[2] Azizzadeh A., Keyhani K., Miller C.C., Coogan S.M., Safi H.J., Estrera A.L. (2009). Blunt traumatic aortic injury: initial experience with endovascular repair. J Vasc Surg.

[3] Tang G.L., Tehrani H.Y., Usman A. (2008). Reduced mortality, paraplegia, and stroke with stent graft repair of blunt aortic transections: a modern meta-analysis. J Vasc Surg.

[4] Chen S.W., Lee K.B., Napolitano M.A. (2020). Complications and management of the thoracic endovascular aortic repair. Aorta (Stamford).

[5] Goswami J., MacArthur T.A., Sridharan M. (2021). A review of pathophysiology, clinical features, and management options of COVID-19 associated coagulopathy. Shock.

[6] Cryer M.J., Farhan S., Kaufmann C.C. (2022). Prothrombotic milieu, thrombotic events and prophylactic anticoagulation in hospitalized COVID-19 positive patients: a review. Clin Appl Thromb Hemost.

[7] Alvarez B., Constenla I., Maeso J., Matas M. (2009). Late thrombosis of a thoracic aorta stent graft: therapeutic management. J Vasc Surg.

[8] Marone E.M., Kahlberg A., Tshomba Y., Logaldo D., Chiesa R. (2012). Surgical conversion for intragraft thrombosis following endovascular repair of traumatic aortic injury. J Vasc Surg.

[9] Marino M., Kasemi H., Martinelli O., Bresadola L., Salvatori F.M., Irace L. (2014). Re-TEVAR for complications after blunt aortic traumatic injury stenting. Cardiovasc Radiol.

[10] Reich H.J., Margulies D.R., Khoynezhad A. (2014). Catastrophic outcome of de novo aortic thrombus after stent grafting for blunt thoracic aortic injury. Ann Thorac Surg.

[11] Abdoli S., Ham S.W., Wilcox A.G., Fleischman F., Lam L. (2017). Symptomatic intragraft thrombus following endovascular repair of blunt thoracic aortic injury. Ann Vasc Surg.

[12] García Reyes M.E., Gonçalves Martins G., Fernández Valenzuela V., Domínguez González J.M., Maeso Lebrun J., Bellmunt Montoya S. (2018). Long-term outcomes of thoracic endovascular aortic repair focused on bird beak and oversizing in blunt traumatic thoracic aortic injury. Ann Vasc Surg.

[13] Liesdek O.C.D., Jacob K.A., Vink A., Vermeulen M.A., Hazenberg C.E.V.B., Suyker W.J.L. (2019). Surgical treatment of acute thoracic Stent graft occlusion. Ann Thorac Surg.

[14] Hostalrich A., Canaud L., Ozdemir B.A., Chaufour X. (2019 Summer). Severe thoracic aorta stenosis after endovascular treatment of blunt thoracic aortic injury. Semin Thorac Cardiovasc Surg.

[15] Martinelli O., Malaj A., Faccenna F. (2020). Open conversion for recurrent endograft occlusion after endovascular treatment of blunt traumatic aortic injury: a peculiar case report. Ann Vasc Surg.

[16] Beijer E., Scholtes V.P.W., Truijers M., Nederhoed J.H., Yeung K.K., Blankensteijn J.D. (2021). Intragraft obstructive thrombus two years after endovascular repair of traumatic aortic injury: a case report and review of the literature. EJVES Vasc Forum.

[17] Chiu M.H., Kaitoukov Y., Roze des Ordons A. (2022). Late Stent thrombosis in a patient with endovascular aortic repair for blunt thoracic aortic injury. Case Rep Vasc Med.

[18] Gorog D.A., Fayad Z.A., Fuster V. (2017). Arterial thrombus stability: does it matter and can we detect it?. J Am Coll Cardiol.

[19] Tan B.K., Mainbourg S., Friggeri A. (2021). Arterial and venous thromboembolism in COVID-19: a study-level meta-analysis. Thorax.

